# An Overview of Power Electronics Applications in Fuel Cell Systems: DC and AC Converters

**DOI:** 10.1155/2014/103709

**Published:** 2014-11-12

**Authors:** M. S. Ali, S. K. Kamarudin, M. S. Masdar, A. Mohamed

**Affiliations:** ^1^Fuel Cell Institute, Universiti Kebangsaan Malaysia (UKM), 43600 Bangi, Selangor, Malaysia; ^2^Department of Chemical and Process Engineering, Universiti Kebangsaan Malaysia (UKM), 43600 Bangi, Selangor, Malaysia; ^3^Department of Electrical, Electronic and System, Universiti Kebangsaan Malaysia (UKM), 43600 Bangi, Selangor, Malaysia

## Abstract

Power electronics and fuel cell technologies play an important role in the field of renewable energy. The demand for fuel cells will increase as fuel cells become the main power source for portable applications. In this application, a high-efficiency converter is an essential requirement and a key parameter of the overall system. This is because the size, cost, efficiency, and reliability of the overall system for portable applications primarily depend on the converter. Therefore, the selection of an appropriate converter topology is an important and fundamental aspect of designing a fuel cell system for portable applications as the converter alone plays a major role in determining the overall performance of the system. This paper presents a review of power electronics applications in fuel cell systems, which include various topology combinations of DC converters and AC inverters and which are primarily used in fuel cell systems for portable or stand-alone applications. This paper also reviews the switching techniques used in power conditioning for fuel cell systems. Finally, this paper addresses the current problem encountered with DC converters and AC inverter.

## 1. Introduction

Renewable energy systems offer environmental and economic advantages in producing energy compared with the conventional fossil fuel systems. Among all types of green energy applications, fuel cells are the most popular because they can provide a continuous power supply throughout all seasons as long as fuel is provided. In comparison, other types of green energy such as solar or wind energy are dependent on the weather conditions. However, the renewable energy supplied by fuel cells has a low-voltage output characteristic, and for any potential practical application, a high step-up DC-DC converter is required [1, 2]. Every source of energy normally comes with a set of challenges in terms of efficiency optimisation, ramifications on the environment, and utilisation. The characteristic electrical output of fuel cells has some drastic shortcomings and a low output voltage that decreases as the load current increases. Fuel cell stacks are also unable to meet transient electrical power demands to the load. However, the low emission and high efficiency of the fuel cell make them a favourable choice for energy sources in portable applications [3, 4].

Implementing power electronics applications in fuel cell systems is a solution that allows fuel cell technology to be used in any application. A fuel cell system can be used in any application with the right selection of power electronics circuits. Using different switching components and switching topologies in a power electronics circuit will yield different results and efficiencies. With the advances in technology made in the semiconductor industry, hard switching power electronics components were added to improve reliability and efficiency. The size of switching components is becoming smaller, which makes them cost-effective. However, there is still a large amount of electrical noise present. The idea behind using a soft-switching method is purposely to reduce noise and switching losses. In addition, switching losses can be reduced by implementing zero-voltage switches (ZVSs) or zero-current switches (ZCSs) [5]. This paper presents a review of power electronics applications in fuel cell systems, and it includes various topologies combinations of DC converters and AC inverters, which are primarily used in fuel cell systems for portable or standalone applications. This paper also reviews the switching techniques used in power conditioning for fuel cell systems.

## 2. Current Technology behind the Main Topology of DC Converters

Several DC converters are available that can increase or decrease the magnitude of the DC voltage and/or invert its polarity. The switch is realised using a power MOSFET and diode; however, other semiconductor switchers, such as IGBTs, BJTs, or thyristors, can be used according to the application [7]. Besides, many semiconductor switchers offered which greatly reduce the switching losses are available in market such as ultra-fast 1200 V IGBTs which can reduce switching and conduction losses with renewable energy technologies focusing on delivering the highest efficiency and reliability. These 1200 V Trench IGBTs are designed to meet the most demanding of any system performance requirement which has been optimized for lowest switching losses and smoothest turn-off in higher frequency. New approaches of the Solderable Front Metal (SFM) technology greatly extend power cycling capability while dual-sided cooling further reduces power dissipation to provide a highly efficient solution [8]. Another solution is Silicon Carbide (SiC) known as SiC-based power electronics which can reduces the size and switch losses in power system by 50% focussing especially on the high power electronics application such as power utilities, smart grids, high-power industrial drive, and renewable energy panel [9].

### 2.1. DC-DC Boost Converter

**Figure 1:**
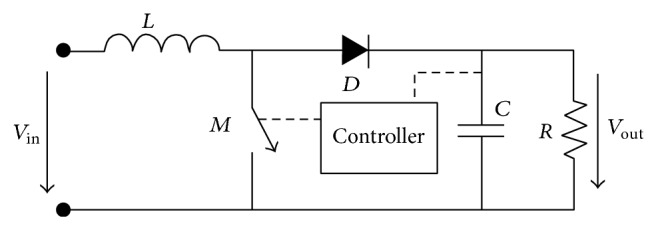
DC-DC boost converter topology, Brey et al. [6].

Basically, a boost converter consists of switching element (*M*), a diode (*D*), an inductor (*L*), and a switching controller, as shown in Figure 1. The switching element is switched between the “on” and “off” state by the controller to boost the input voltage to the desired output voltage. During the “on” state of the switching element, electrical energy is stored in the inductor, and then the capacitor supplies current to the load and the diode with a reverse bias. When in the “off” state, stored energy is transferred to the load and capacitor through the diode [6]. The boost converter operates in one of two modes, continuous-conduction mode or discontinuous-conduction mode, which is characterised by the current waveform of the inductor [10]. The inductor current is greater than zero all the time when in continuous-conduction mode, and the inductor current falls to zero after each switching cycle when in discontinuous-conduction mode [6].

The boost converter has characteristics such as a continuous input current, a low input ripple current, and good clamping of output diodes requiring less than half of the challenging task as it invariably involves the analysis of various voltage ratings of rectifier diodes, which are ideal for fuel cell applications, and many attempts have been made to improve the efficiency of the boost topology at low input voltages, making it the ideal choice for fuel cell applications. These improvements include optimising the transformer design to achieve very low leakage inductances, taking advantage of modern power electronics switching, and eliminating the need for a voltage clamp circuit [12–19].

Yang et al. [20] proved that a new quadratic boost converter with an additional capacitor-inductor-diode (CLD) cell has better characteristics than the conventional quadratic boost converter and that the topology is suitable for extreme high-voltage step-up ratio applications. Yao et al. [21] analysed the full-bridge (FB) boost converter that combines the two-edge-modulation (TEM) scheme and the FB cell that is leading-edge modulated with a three-mode PS-TEM control scheme used to improve the efficiency and reliability. Apparently, they proved that the operation of the FB-boost converter and three-mode dual-frequency PS-TEM control are more effective, while Hwu and Yau [22] demonstrated the combination of a KY boost with a traditional synchronously rectified (SR) boost converter for low-ripple applications and found that the efficiency is 90% or more above the half-load point.

Park et al. [24] and Bo et al. [25] proposed a new soft-switching boost converter using a soft-switching method with a resonant inductor and capacitor, an auxiliary switch, and diodes and proved that the converter reduces switching losses more than a conventional hard-switching converter and that the efficiency increases approximately 96%. Ivanovic et al. [26] developed new control algorithms for higher converter efficiency that require loss model parameters that can be used in the algorithm to improve the efficiency of the boost converter.

### 2.2. DC-DC Buck Converter

The buck converter is used to reduce the DC voltage and has a conversion ratio of *M*(*D*) = *D*. It is widely used because of its simple topology, which is characterised by a low number of components, low control complexity, and no isolation. In the conventional buck converter, only a single active switch is used, and the maximum voltage applied across the terminals of the semiconductors equals the input voltage obtained using the hard switching technique. However, this conventional method produces low efficiency because of the high conduction losses due to high-voltage-rated devices and high switching losses. Buck converters can be used in low-power-range regulators and very high range step-down converters [27, 28].

Rodrigues et al. [29] presented a study of a DC-DC buck converter with three-level buck clamping, zero-voltage switching (ZVS), active clamping, and constant-frequency pulse width modulation (PWM) and proved that the voltages across the switch are 50% lower compared with a two-level ZVS buck-buck converter. Chen et al. [30] proposed a new single-inductor quadratic converter using average-current-mode control without slope compensation, which minimises several power management problems, such as efficiency, EMI, size, transient response, design complexity, and cost. Jahanmahin et al. [31] proposed an improved configuration for DC-DC buck and boost converters, which is a novel method for increasing output power by utilising two storage elements and reducing the output ripple voltage for buck and boost converters.

### 2.3. DC-DC Buck-Boost Converter

**Figure 2:**
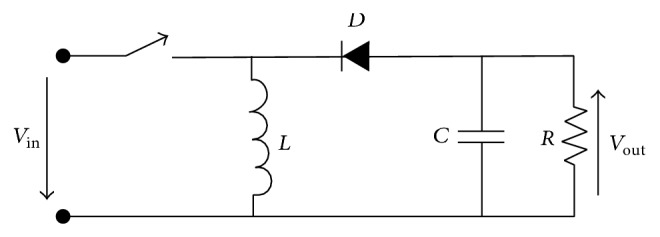
DC-DC buck-boost converter topology, Brey et al. [6].

**Figure 3:**
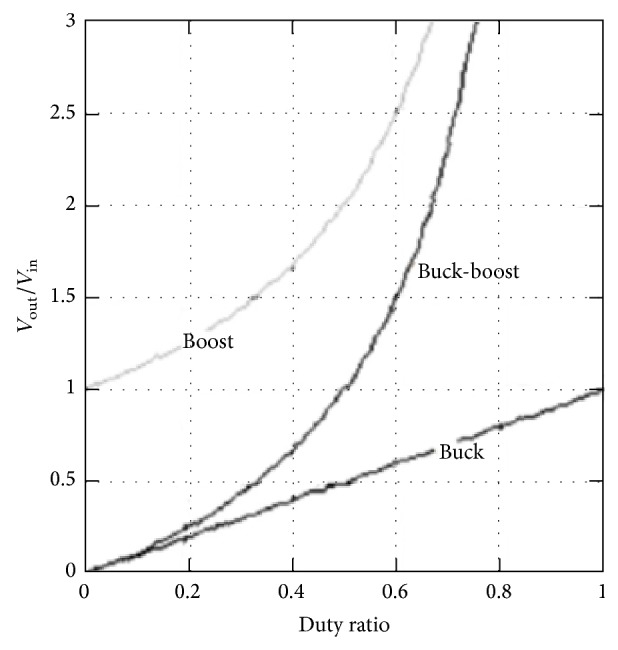
Comparison of duty ratio in buck, boost, and buck-boost converter, Brey et al. [6].

The basic circuit of a buck-boost converter consists of a switching element, inductor, diode, and capacitor. The difference between a buck-boost and a boost converter is the arrangement of the switching element placed before the inductor, as shown in Figure 2. The buck-boost topology can produce an output voltage that is equal to, less than, or greater than the input voltage. The buck-boost topology is suitable for portable applications, which require a wide range of output levels, and it is an attractive choice when a large current is supplied [6]. The output voltage is equal to the input voltage when the duty cycle is 0.5. When the duty cycle is less than 0.5, the buck-boost converter operates in buck mode, causing the output voltage to be lower than the input voltage. To make the buck-boost converter operate in boost mode and cause the output voltage to be higher than the input voltage, the duty cycle must be greater than 0.5. This relationship is shown in Figure 3 [6].

Chen et al. [32] proposed a buck-boost PWM converter having two independently controlled switches that can work as a boost or as a buck converter depending on the input-output conditions; this approach puts lower stresses on the components. Hwang et al. [33] proposed a low-voltage positive buck-boost converter using an average current controlled with a simple compensation design. This approach can reduce some power management problems, such as size, cost, design complexity, and a simple compensation design, and it provides regulated output with a maximum efficiency of 72% at a switching frequency of 1 MHz. Boopathy and Bhoopathy Bagan [34] presented a novel method of implementing a real-time buck-boost converter with an improved transient response for low-power portable applications and significantly improved the efficiency from 16% and 19%. Hwu and Peng [35] proposed a novel buck-boost converter combining KY and buck converters, which can solve the problem of voltage bucking of the KY converter and increase the application capability of the KY converter with an efficiency above the DC load current of 0.25 A of 88% or more.

### 2.4. DC-DC Cuk Converter

The Cuk converter contains an inductor in series with the converter input and output port. The switch network alternately connects a capacitor to the input and output inductors. The conversion ratio *M*(*D*) is identical to that of the buck-boost converter. Hence, the converter also inverts the voltage polarity, while either increasing or decreasing the voltage magnitude. The Cuk converter is a modified boost-buck converter and can be used either to step up or step down the output voltage with respect to the input. It produces a negative output voltage from a positive input voltage, and its advantage is the presence of both input and output inductors. These inductors lower the current ripple on the input source and the load. Furthermore, the Cuk converter also has a higher efficiency, reduced EMI generation, and a better dynamic response [36]. Lin et al. [37] designed and demonstrated an active snubber zero-voltage switching Cuk converter for achieving parallel operation and balanced current sharing.

### 2.5. DC-DC SEPIC Converter

The single-ended primary inductance converter (SEPIC) can either increase or decrease the voltage magnitude. However, it does not invert the polarity. The conversion ratio is *M*(*D*) = *D*/(*D* − 1). The SEPIC has the features of the buck-boost operating mode, no polarity inversion, low input current pulsation, and a wide input voltage range. It also has the following advantages, which are applied to the electricity generation system of the fuel cell: (i) It is a converter that can operate under boost or buck situations, and there is greater elasticity for assisting the design of the auxiliary source. (ii) Compared with other common boost or buck converters, the SEPIC converter has no issues with an inverse polarity output voltage. (iii) The input terminal of a SEPIC converter contains an inductor, which can reduce the input current pulses and overcome the disadvantage of the electric current pulses of the fuel cell to increase the accuracy of the control [38]. The SEPIC is commonly used in light emitting diode (LED) backlights and photovoltaic applications because it can produce noninverting output and also can operate as a step-up and step-down converter. However, its power conversion efficiency is lower than that of other converters, such as the buck and boost converter, because its extra inductor and capacitor cause additional power losses [39–41]. Song et al. [42] proposed a modified SEPIC design for a step-up and step-down converter having higher power conversion efficiency than the original SEPIC topology in continuous conduction mode (CCM). The input power can be directly delivered to the output with few losses.

## 3. Application of DC Converters in Fuel Cell Systems

Due to the limitations of fuel cells, which include low voltages, low current densities, and unstable power production, the DC converter has become the most important component in fuel cell systems for portable or stand-alone applications. Normally, a single DMFC can supply only approximately 0.3–0.5 V under loaded conditions. By using a DC converter, these limitations can be solved with a converted voltage source from the fuel cell [1]. Various DC converters have been developed to support fuel cell systems, but the efficiency of a DC converter depends on the conduction and switching losses. If there are fewer conduction and switching losses, then the DC converter operation is more efficient. By reducing the number of components used and their operating ranges, the conduction losses can be effectively reduced [43]. A fuel cell system application is necessary to use a power management circuit to generate the desired load voltage level. Therefore, it may require a nominal supply voltage, which can be above, below, or equal to the fuel cell generating voltage [6]. Therefore, the system designed for the nominal supply voltage will require a converter capable of stepping up or stepping down the fuel cell voltage [44]. There are two converters widely used in fuel cell systems for the single-stage level or for low-voltage portable applications to step up and step down the fuel cell voltage with a high efficiency: boost converters (step-up) and buck converters (step-down).

### 3.1. Single-Stage Topologies

To fulfil the operational requirements of fuel cell systems, the researchers developed various topologies for single-stage conversion either using a DC-DC converter or a DC-AC inverter. The voltage generated from the fuel cell can be converted directly into a regulated DC voltage, or it can be converted directly into an AC voltage as a supply voltage, depending on the AC or DC load. A single-stage topology has reduced component counts and is simple to control.

Brey et al. [6] tested several DC converters in their study on the power conditioning of fuel cell systems in portable applications and claimed that the DC-DC boost converter gives the best performance for a 100 W fuel cell power condition supplying the desired regulated output voltage level and maintaining an input current ripple below 2% of the nominal input current. Nymand et al. [46] presented a comprehensive comparison between buck and boost topologies and claimed that the boost converter topology is more appropriate for low-voltage fuel cell applications. Kui-Jun and Rae-Young [47] proposed a zero-voltage-transition (ZVT) two-inductor boost converter using a single resonant inductor with a maximum efficiency at 92.4% due to the ZVS operation under the *V*_in_ = 30 V condition.

**Figure 4:**
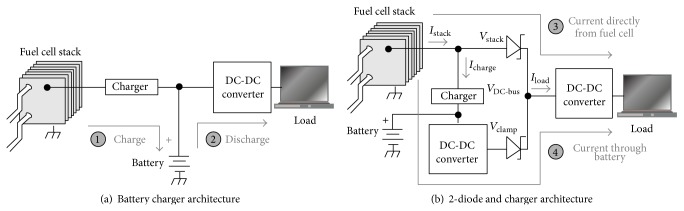
Power control scheme for fuel cell and battery hybrid, Chang et al. [11].

Chang et al. [11] proposed a complete hybrid active DMFC system design by implementing a hybrid between the DMFC stack and a Li-ion battery to share the applied load to exploit the high energy density of the fuel cell and the power density of the battery, as presented in Figure 4. A conventional battery charger hybridisation circuit relies on the battery as a primary power source, as shown in Figure 4(a). The DMFC stack operates only when charging the battery meaning that there are no direct supplies from fuel cell to laptop. The system operates starting with the DMFC stack by charging the battery first, and then the battery will supply power to DC-DC converter to operate the laptop. Then for Figure 4(b), there to ways how the fuel cell powered the laptop either direct supply from fuel cell to DC-DC converter and to the laptop or from fuel cell charging the battery first then battery supply power to DC-DC converter to operate the laptop. These two operation ways occurred simultaneously. Supplied power from battery will become additional current for the system to back up a current to another direct supplied from fuel cell. This additional current goes through three converters: the battery charger, the battery output converter, and the output regulation converter.

Harfman-Todorovic et al. [49] proposed a hybrid DC-DC converter for a fuel cell-powered laptop computer. However, this power distribution system has problems with a large reduction of voltage, for which it needs a voltage regulator module (VRM). Alternative power distribution architecture with difference interfacing energy sources is presented, and the hybrid converter proved the capability of interfacing a DC-link with multi-input power sources in a 30 W fuel cell.

### 3.2. Multistage Topology

Power conditioning combinations involving two types of DC-DC converters or an AC inverter are recognised as multistage topology. In this topology voltage or current of the fuel cell will convert first to desired value by using DC-DC converter. This DC-DC converter may involve any type of DC-DC converter such as boost, buck-boost, and so on to get desired value of voltage or current becoming input variable to the inverter and is also used to charge ultra-capacitor or battery. Second step in this topology is DC voltage from DC-DC converter inverted to AC voltage. The disadvantage of this topology using more components compared with a single stage will make the power loss in a multistage topology greater than that in a single-stage topology.

Zhu et al. [50] proposed a hybrid energy system topology between DMFC and a supercapacitor in which the DMFC and the super capacitor bank are connected to a common DC voltage bus through a boost converter and a bidirectional converter. A similar topology has been proposed and discussed in detail by others [1, 41, 42]. Here, a supercapacitor is used to balance the system's power flow for load voltage regulation by delivering the deficit power during heavy load conditions and absorbing the surplus power from the DMFC during light load conditions while the DMFC is constantly tracked at the MPP by the MPPT controller. The supercapacitor can be charged and discharged rapidly under varying load conditions for an uninterrupted power flow to the load with suitable design of the bidirectional converter.

**Figure 5:**
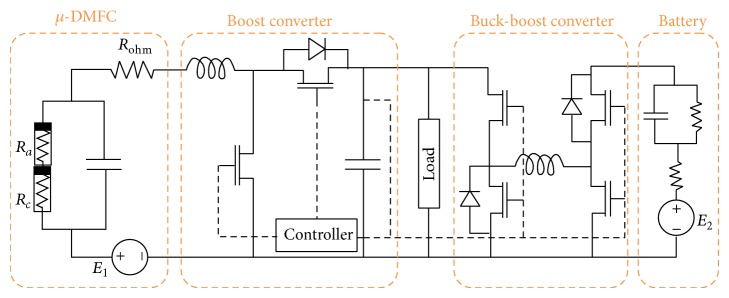
The hybrid power system (small-scale DMFC and Li-ion battery), Alotto et al. [23].

Kwon et al. [51] proposed a high-efficiency active DMFC system for portable applications. This system used a smart battery to support the fuel cell system that consisted of two buck-boost converters to increase the power conversion efficiency. Alotto et al. [23] claimed that the hybrid sources can combine the high power density of batteries with the high energy density of fuel cells to improve the runtime of portable electronic devices, as shown in Figure 5, in which the synchronous boost converter is connected after the fuel cell to boost the voltage from the fuel cell up to the DC-bus voltage (typically 3.3 V for electronic devices). In a real application, the fuel cell will be connected in series to attain acceptable input voltage levels for a full load (2–2.4 V), while on the battery side, the H-bridge buck-boost converter will be employed to manage charging and discharging the battery. This H-bridge buck-boost converter is emerging with an analogue design, which means this converter can be operated as a buck converter when the battery is fully charged or as a boost converter when the battery is almost discharged.

## 4. Limitations of Current DC Converters

**Figure 6:**
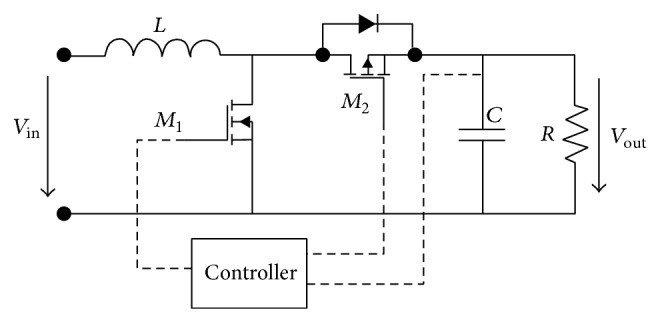
Synchronous DC-DC boost converter topology, Brey et al. [6].

Boost converters have a voltage collapse point when the output power is too high and the input current becomes excessive, leading to high losses, which in turn reduces the efficiency further, requiring even more input current, causing voltage collapse. There are also large voltage drops across the diode. For low-voltage applications, replacing the diode with a switching element can improve the efficiency of the converter, which results in a synchronous DC-DC boost converter topology, as shown in Figure 6. In this circuit, a time delay must be used to prevent a shoot-through current between turning off *M*_1_ and turning on *M*_2_. In this way, the voltage drop across *M*_2_ is the lowest, and the efficiency can be improved [6]. Two power management schemes were used to achieve a high efficiency within the whole load range: PWM control for the heavy load condition and PFM control for the light load condition [52, 53]. The converter has different power losses (conduction loss and switching loss) under different load conditions.

The principal disadvantage of a boost converter is the high switching noise. This noise is generated when turning on and off the switching element, and it deteriorates the quality of the output voltage and degrades the performance of the fuel cell. Several solutions to avoid this problem have been proposed: a snubber circuit can be placed at every switch, additional electromagnetic interface at the input and output of the converter can be incorporated, and the soft switching technique can be used [6].

**Figure 7:**
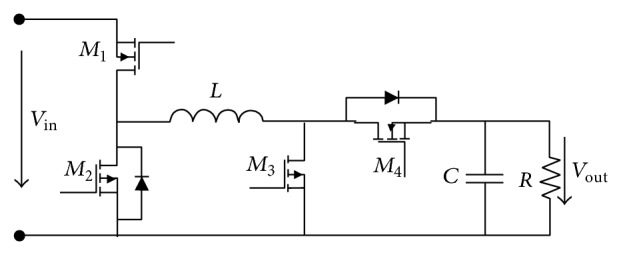
Noninverting buck-boost converter topology, Brey et al. [6].

The buck-boost converter requires a greater duty cycle than the boost converter to boost the input voltage at the same output voltage level. This causes the buck-boost converter to be less efficient compared with the boost converter because higher duty cycles increase conduction losses in the switching element. Another disadvantage is that the obtained output voltage polarity is opposite to the supply voltage, meaning this topology is not suitable for some applications. To solve this problem, a noninverting buck-boost converter can be used [6]. Noninverting buck-boost converter topology involves cascading between a buck converter and a boost converter, as shown in Figure 7. For low-voltage applications, a switch can replace the diode to improve the efficiency. This topology has the same problem with switching noise as the boost converter topology [6].

One of the biggest goals in power conditioning design is to operate the converter with a high switching frequency to minimise both the cost and size of the respective power conditioning and also to optimise the efficiency. Conventional hard-switched pulse-width modulation (PWM) suffers from high switching losses, high device stress, and objectionable EMI when operating at a high switching frequency compared with the soft-switching PWM technique, which is being used for high switching frequency operation with high efficiencies and large power-to-volume ratios [45].

## 5. Recent Development in DC-DC Converters 

**Figure 8:**
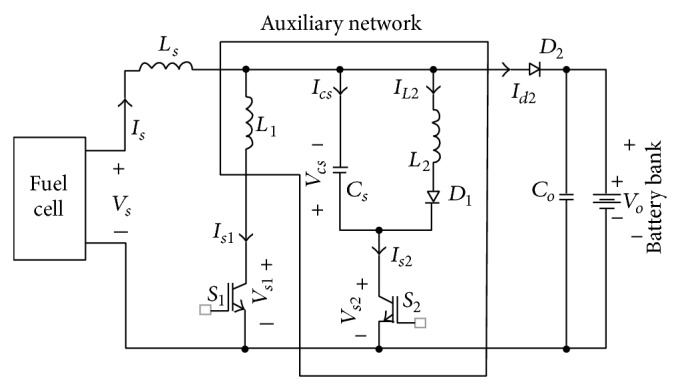
New soft-switched DC-DC boost converter, Saha [45].

**Figure 9:**
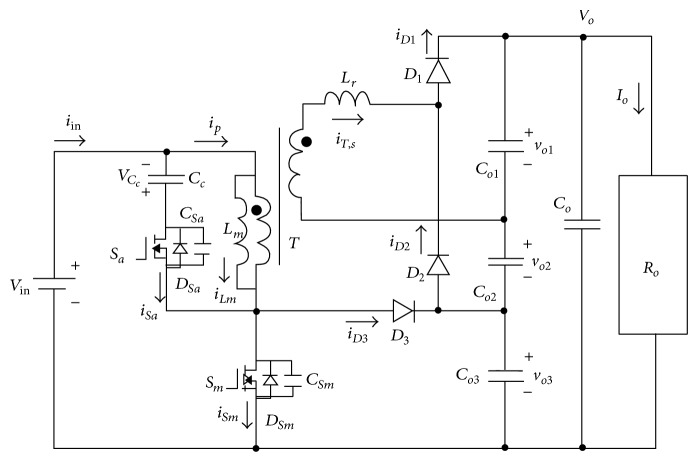
New zero-voltage switching DC-DC converter, Lin and Dong [48].

Saha [45] proposed that the efficient soft-switched boost converter can be increased by using an auxiliary network in addition to the boost inductor *L*_s_, boost switch *S*_1_, and boost diode *D*_2_ as shown in Figure 8. The auxiliary network consists of on-switch element *S*_2_, one diode *D*_1_, two inductors *L*_1_, *L*_2_, and one snubber capacitor *C*_s_. This converter operates under seven modes, and it was determined that the converter can operate with a high efficiency (above 95%) at full load. The output voltage of this converter can be regulated by varying the pulse-width of the main switch *S*_1_. Lin and Dong [48] proposed a new zero-voltage switching DC-DC converter for renewable energy conversion systems based on a boost converter and a voltage-doubler configuration with a coupled inductor to achieve a high step-up voltage conversion ratio, as shown in Figure 9. In this configuration, an active snubber is adopted to clamp the voltage stress of the active switch and to release the energy stored in leakage and magnetising inductances. In conventional boost converter, the adopted converter has a wide turn-off period to achieve high voltage. By adopting an asymmetrical pulse-width modulation to control active switches, leakage inductance and output capacitance of the active switch are resonant in the transition interval. The result from this configuration is that both switchers turn on at zero-voltage switching which overcome the problem of the conventional boost converter with low circuit efficiency and narrow turn-off period. Al-Saffar et al. [54] proposed a new soft-switched pulse-width modulation (PWM) quadratic boost converter that is suitable for application to a wide fluctuating DC input voltage range. The voltage gain of the conventional PWM DC-DC boost converter has limitations for practical applications, even under extreme duty cycle conditions, due to parasitic components [55]. A new soft-switched pulse-width-modulated (PWM) quadratic boost converter that is suitable for applications with a wide fluctuating DC input voltage range is designed for fuel cell systems. The efficiency of this converter is equal to 92.3%. Finally, Delshad and Farzanehfard [56] proposed a new zero-voltage switching current-fed push-pull DC-DC converter. The auxiliary circuit in this method is introduced purposely to provide a zero-voltage switching condition and also to absorb the voltage surge across the switches at the turn-off instance. Therefore, the size and weight of the converter can be reduced, and the efficiency of the converter can be increased. To reduce the size and weight of the converter, high-frequency operation is required to reduce the size of the induction element and other reactive components. This converter controls via PWM with a very simple implementation control circuit. This converter can be operated at higher frequencies, and these frequencies are higher than the frequency of a conventional current-fed converter. In this converter, the clamp circuit not only absorbs the voltage surge across the power switches but also provides soft switching conditions for all semiconductor devices. Because the converter uses only one input filter inductor and it does not use any clamp winding, the cost and size can be reduced compared with conventional converters.

## 6. Conclusions

The DC converters with various topologies contribute to the use of renewable energy in various applications, especially in portable or stand-alone applications. A review on DC converters shows that they can be used to produce a more efficient conversion of power from the fuel cell to the load. Using a DC converter or a combination of DC converters can address the limitations of fuel cell, which include unregulated voltage, low voltage, low current density, and unstable power. A hybrid DC converter with a battery or a super capacitor or other external supplies can stabilize the power conditioning to balance the excess and insufficient power condition in the fuel cell. This review also shows that the switching technique is the main element of a DC converter. Introduction of the soft-switching operation to DC converters introduces improvements in terms of increased converter power density and converter efficiency. The topology of the DC converter in the power conditioning unit can be divided into single-stage and multistage topologies. In single-stage topologies, the DC converter stands alone, but in multistage topologies, the DC converter is combined with DC converters or with AC inverters.

In conclusion, the design of DC converter topologies is considered important in fuel cell systems. Therefore, more studies on the development of new topologies for DC converters, including new switching techniques, are needed to create a higher efficiency and improve the existing switching technique. For specific applications of portable fuel cell systems, the size and energy density are considered very important. Currently, portable fuel cell focuses on two types of fuel cells that can fulfil the size and energy density requirements: direct methanol fuel cells (DMFCs) and direct borohydride fuel cell (DBFCs). Fuel cells such as DMFCs and DBFCs with improved power converter technologies can be considered as promising alternative energy sources for portable applications.
